# Doping Use in High-School Students: Measuring Attitudes, Self-Efficacy, and Moral Disengagement Across Genders and Countries

**DOI:** 10.3389/fpsyg.2020.00663

**Published:** 2020-04-28

**Authors:** Laura Girelli, Elisa Cavicchiolo, Fabio Alivernini, Sara Manganelli, Andrea Chirico, Federica Galli, Mauro Cozzolino, Fabio Lucidi

**Affiliations:** ^1^Department of Human, Philosophical, and Educational Sciences, University of Salerno, Salerno, Italy; ^2^National Institute for the Evaluation of the Education System (INVALSI), Rome, Italy; ^3^Department of Social and Developmental Psychology, Sapienza University of Rome, Rome, Italy

**Keywords:** high-school students, self-regulatory efficacy, moral disengagement, measurement invariance, gender differences, cross-cultural differences

## Abstract

The main aim of this research was to test the factorial validity and measurement invariance across genders and countries of a set of instruments designed to assess high-school students’ attitudes, self-regulatory efficacy, and moral disengagement with regard to doping. A second aim was to examine the criterion and predictive validity of these scales. In total, 402 high-school students from Italy, Romania, and Turkey (40.0, 25.1, and 34.9%, respectively; *M* age 14.78 years old; SD = 1.04; 52.8% females) completed questionnaires measuring attitudes toward doping, self-regulatory efficacy in refraining from doping, doping-specific moral disengagement, and intention to use doping substances. A confirmatory factor analysis (CFA) supported our expectations with regard to the factor structure of the scales. Multigroup CFAs provided evidence for the full equivalence of the measures across males and females and partial equivalence of the measures across the three countries. The results of the latent mean comparison showed that male students had lower levels of self-regulatory efficacy than females and that Romanian and Turkish students had higher levels of moral disengagement and lower level of self-regulatory efficacy than Italian students. Finally, the results of a structural equation modeling supported the hypothesis that the proposed model predicted students’ intentions to use doping, thus generally confirming the criterion and the predictive validity of the measures. These findings suggested the validity of a set of instruments measuring attitudes toward doping, self-regulatory efficacy to refrain from doping, and doping-specific moral disengagement in high-school students from a cross-gender and a cross-cultural perspective and provided meaningful estimates of the differences in the three factors between males and females as well as between Italian, Romanian, and Turkish high-school students.

## Introduction

The use of doping is recognized as a relevant issue in sport. A growing body of literature indicates that not only elite athletes use and abuse doping substances but also those who engage in amateur and recreational sports (Dunn and White, 2011) sometimes to an even greater extent than professional athletes (Wanjek et al., 2007). For this reason, doping has been identified as a rising public health problem. Furthermore, a rise in doping has been detected in the young, whether they are athletes or not (LaBotz and Griesemer, 2016), a tendency which is becoming apparent at increasingly young ages (LaBotz and Griesemer, 2016; Nicholls et al., 2017). The term “doping” generally indicates the use of illegal performance- and appearance-enhancing substances (PAES; Mallia et al., 2013), but several studies in reference to various different sports have demonstrated the extensive consumption of legal PAES, such as proteins, amino acids, creatine, etc. (Bell et al., 2004). Even though these substances are legal, they may act as a “gateway” to doping practices (Lucidi et al., 2017). Also the use of legal PAES appears to be increasing among young people (Bell et al., 2004; Hoffman et al., 2008; LaBotz and Griesemer, 2016). For example, a study conducted in the US revealed that, among high-school students, 38.8% of boys and 18.2% of girls reported protein supplement use; furthermore, although students who regularly practiced sports used these substances more frequently, also 18.2% of other students frequently consumed protein supplements (Eisenberg et al., 2012). Therefore, for both legal and illegal PAES, high-school students are one of the groups that are more at risk (Dodge and Hoagland, 2011; Dunn and White, 2011; Eisenberg et al., 2012).

A growing body of research has investigated the factors that affect the use of doping in athletes and non-athletes (Lucidi et al., 2008; Petróczi and Strauss, 2015; Mallia et al., 2016; Kavussanu et al., 2019). A recent meta-analysis identified positive attitudes toward doping, morality, and self-efficacy to resist from doping as being some of the strongest psychological predictors of doping intentions and behaviors (Ntoumanis et al., 2014). Although these factors have been extensively investigated in the context of doping in competitive sport, so far, no studies have specifically examined the measurement invariance of these scales across genders and across countries in a population of non-athletes. Valid instruments, which are equivalent across males and females and across countries, are essential in order to make the prediction of doping intention and behavior more accurate. In the subsequent paragraphs, we will define the constructs under examination. We will then outline the importance of possessing valid instruments for measuring them in order to facilitate doping prevention.

According to the Theory of Planned Behavior (TPB; Ajzen, 1991), one of the leading socio-cognitive theories, the term “attitude” refers to the degree to which individuals have a favorable or unfavorable evaluation of a behavior. One’s attitude toward doping therefore consists of a positive or negative evaluation of its use either for performance enhancement or for esthetic reasons. Research conducted on the basis of the TPB has demonstrated that attitudes toward doping are effective in predicting doping intentions and behavior (Lucidi et al., 2004, 2008; Wiefferink et al., 2008; Goulet et al., 2010; Lazuras et al., 2010; Mallia et al., 2016). This applies to various different groups, such as elite athletes (Lazuras et al., 2010), non-professional athletes (Wiefferink et al., 2008), and students (Lucidi et al., 2008; Zelli et al., 2010; Mallia et al., 2013). These results are therefore generalizable across different populations and contexts.

According to the Social Cognitive Theory (Bandura, 1997), “perceived self-efficacy” refers to the beliefs that individuals hold about their capacity to achieve their personal goals and to overcome difficulties. According to Bandura, self-efficacy must be tailored to the particular domain of functioning or conduct that is being investigated (Bandura, 1997). Thus, as in the context of doping, social normative pressures, such as the influence of significant others, may have a significant role with respect to the use/abuse of illegal substances (Kindlundh et al., 1999), individuals’ beliefs about their own ability to resist to them are fundamental. Therefore, “doping-specific self-regulatory efficacy” refers to one’s ability to resist social pressure toward doping and to avoid or cope with situations in which doping occurs more often. Studies have shown that, in addition to attitudes, self-regulative efficacy toward doping is effective in predicting doping intentions and self-reported doping use (Lucidi et al., 2008; Lazuras et al., 2010; Zelli et al., 2010; Barkoukis et al., 2013; Mallia et al., 2013, 2015).

Within the Social Cognitive Theory (Bandura, 1991), a construct that has recently been receiving increasing attention is that of moral disengagement (Kavussanu et al., 2016), which is a process of convincing oneself that ethical standards do not apply in a particular context, by suspending or deactivating the mechanism of self-condemnation and self-sanction. One’s internal moral standards can thus be activated or inhibited by mechanisms of self-justification. In these cases, people may not feel obliged to make decisions that conform to their normal moral standards. Moral disengagement in the context of doping refers to the “self-serving self-regulatory process that allows people to dope while still believing they are acting morally” (Lucidi et al., 2017, pp. 2). It constitutes a moral justification for doping, for example, by comparing it with more extremely inhumane actions or when substance use is not perceived as being under the individual’s own control. Many studies have demonstrated that moral disengagement influences adolescents’ intention to use doping and doping substances effective use (Lucidi et al., 2008; Kavussanu et al., 2016).

Despite the fact that measures of attitudes, self-regulatory efficacy, and moral disengagement toward doping have been used extensively in research into doping (Kavussanu et al., 2016, 2019; Mallia et al., 2016; Lucidi et al., 2017) and much support has been found for their internal consistency and reliability, no studies have been published that establish the validity of these scales for non-athletes. This is important since various studies have shown that the use of illegal PAES is a widespread issue (Mallia et al., 2013), which is not limited to athletes and is particularly relevant for adolescents (Barkoukis et al., 2016). Moreover, the use of doping substances poses a significant threat in many other area of adolescents’ lives, as it has been associated with behaviors that pose a high health risk, such as the abuse of alcohol and illicit drugs (e.g., DuRant et al., 1995; Kindlundh et al., 1999), and it is often seen as having an effect on variables related to young people adjustment, such as academic achievement (DuRant et al., 1995; Kindlundh et al., 1999). It is therefore particularly important to prevent doping in adolescents because at this age, individuals are more susceptible to normative influences (Ntoumanis et al., 2014) and their attitudes are shaped. According to various studies (Smith and Stewart, 2010; Barkoukis et al., 2016), our values with regard to sports, as well as doping, are associated with social norms that shape our attitudes toward the use of doping substances. It is therefore highly recommended to support interventions targeting adolescents’ perceptions of sport values, social norms, and attitudes toward PAES use in sports. Interventions which aim to improve adolescents’ perceptions of sporting values, social norms, and attitudes toward the use of PAES in sports therefore need to be supported. For this purpose, we need to possess valid instruments for the screening and evaluation of interventions focused on combating doping among young people.

In addition, no study hitherto has analyzed the measurement invariance of these instruments across genders and countries. Doping is influenced by the interplay of several factors, such as personal characteristics and social contexts (e.g., social norms) (Ntoumanis et al., 2014) that can vary for several reasons (e.g., different countries, laws, or individual backgrounds). In order to evaluate and compare anti-doping interventions, it is therefore important to use measures that can be applied regardless of the context or individual-specific characteristics. Limited studies have investigated the validity of several different instruments in a sample of athletes (Kavussanu et al., 2016; Mallia et al., 2016). In particular, Kavussanu et al. (2016) investigated the validity and the measurement invariance across genders of a 12-item measure moral disengagement toward doping which was specifically devised for the context of sport, whereas Mallia et al. (2016) developed and validated measures of self-regulatory efficacy in refraining from doping and moral disengagement toward doping in team contexts, as well as examining the measurement invariance of the scales across different countries. As previously pointed out, the factorial validity of these scales in the population of non-athletes has so far not been analyzed. Furthermore, it is necessary to determine whether doping-related measures of attitudes, self-efficacy, and moral disengagement are valid in terms of their equivalence across males and females and across different cultures. The present study was designed to make up for this lack, and it represents the first assessment of a set of measures of psychosocial determinants of doping use, as well as the first examination of their measurement invariance across males and females and across countries in a sample of non-athletes.

### The Present Study

The aim of this research was to test the factorial validity of a set of instruments measuring attitudes, self-efficacy, and moral disengagement toward doping in a sample of non-athletes and to test the measurement invariance of these scales across genders and countries, i.e., to determine the extent to which individuals of different groups interpret the items of a measure in an equivalent way (Cheung and Rensvold, 2002), a factor that is essential in order to reliably compare these groups. When there is a satisfactory level of measurement invariance, any differences that are detected between the groups reflect genuine differences in the variables and rather than variations in the responses that are merely due to a different interpretation or understanding of the items in the questionnaires. Previous research has indicated the presence of gender differences in attitudes, self-regulatory efficacy, and moral disengagement in several populations, including high-school students (Lucidi et al., 2008) and team sport athletes (Boardley and Kavussanu, 2008). Typically, females report less positive attitudes toward doping, as well as higher levels of self-regulatory efficacy and of moral disengagement than males (Lucidi et al., 2008). Since gender differences are common regarding these factors, it is fundamental to investigate whether the differences measured are simply due to different interpretations of the items, which means that it is necessary to test the measurement invariance of these scales across males and females. Previous studies have also identified cross-cultural differences in these variables in different populations. For example, Mallia et al. (2016) found that German team-athletes considered themselves more able to resist social pressure with regard to doping use than did the Greek and Italian team-athletes, while the Greeks reported that they lowered their personal moral standards to a greater extent than their Italian and German peers. Hence, in order to understand whether different responses between the groups reflect actual differences in the variables examined and not just differing interpretations of the items, it is also necessary to investigate measurement invariance across countries. For this reason, the present investigation included participants from three different countries: Italy, Romania, and Turkey. By comparing latent means, we closely examined the differences within the two groups—one based on gender, the other on nationality—with regard to the measures used. Finally, in order to examine the criterion and the predictive validity of the scales, the present study also examined the associations of the measures of attitudes, self-efficacy, and moral disengagement to participants’ reported intentions to use doping substances in the near future.

## Materials and Methods

### Participants and Procedures

The participants were high-school students (*n* = 402; *M* age 14.78 years, SD = 1.04; age range 14–18 years; 52.8% females) who participated in a project designed to promote life skills in young people in order to promote health. The project involved students from three European countries: Italy (*n* = 161; 40%; *M* age = 13.89 years; SD = 0.88), Romania (*n* = 101; 25.1%; *M* age: 15.28 years, SD = 0.72) and Turkey (*n* = 140; 34.8%; *M* age = 15.47 years, SD = 0.53). In accordance with ethical guidelines, a description of the study with a consent form to be signed was sent to the students’ parents and voluntary participation was only requested from those students whose parents had given their consent. All of the participants were informed about confidentiality and anonymity and their right to withdraw from the study at any time. The participants were invited to complete an online questionnaire lasting about 20 min during classroom hours. All of the questionnaires were anonymous.

### Measures

The questionnaire, based on the instruments developed by Lucidi et al. (2008), included measures of attitudes toward doping, doping-specific self-regulatory efficacy, moral disengagement and intention to use doping substances. As the original versions of the scales were in Italian, all the scales were translated from Italian into Romanian and Turkish using the standardized back translation procedures (Hambleton and Patsula, 1998). Thus, the questionnaire, which had already been translated into Romanian and Turkish by a professional translator, was translated back into the original language (Italian) by another expert translator in order to ensure that the original meaning of the questions had not been changed in any way. Descriptive statistics and reliability coefficients for all the measures used in the study are reported in Table 1. The measures in Italian, Romanian, Turkish, and English are included in the Supplementary Materials.

**TABLE 1:** Descriptive statistics, reliability, and zero-order correlations among all the key variables of the study.

	**Mean (SD)**	**Cronbach’s alpha**	**Zero-order correlations**			
			**(1)**	**(2)**	**(3)**	**(4)**
(1) Attitudes toward doping	1.89 (0.99)	0.82				
(2) Self-regulatory efficacy	5.45 (1.62)	0.85	−0.40**			
(3) Moral disengagement toward doping	2.00 (0.87)	0.72	0.34**	−0.32**		
(4) Intention to use doping substances	1.48 (0.84)	0.82	0.58**	−0.38**	0.41**	

**p < 0.01.

#### Attitudes Toward Doping

The respondents’ attitudes toward doping were measured by five items, with responses on five-point semantic differential scales with the bipolar adjectives: “*useless/useful*,” “*foolish/wise*,” “*undesirable/desirable*,” “*negative/positive*,” “*harmful/beneficial*,” asking participants to express the extent to which the “use of illegal substances to improve sporting performance or physical appearance would be for you…”

#### Doping-Specific Self-Regulatory Efficacy

Self-regulatory efficacy toward doping was measured by six items referring to the extent to which participants felt confident in avoiding or coping with situations or circumstances in which doping use is more likely on a 5-point Likert-type scale ranging from *not at all capable* (1) to *completely capable* (5).

#### Doping Moral Disengagement

The participants’ moral disengagement was measured by six items addressing the mechanisms of moral disengagement that are relevant to doping. For example, the item “compared to the damaging effects of alcohol and tobacco, the use of illicit substances is not so bad” refers to the mechanism of advantageous comparison, while the items “it is not right to condemn those who use illicit substances to improve their body, since many do so” refers to the mechanism of displacing or diffusing responsibility. No items measured the attribution of blame or dehumanization as these processes are not pertinent in the field of doping research (Lucidi et al., 2008). For each item, students rated their agreement on a five-point Likert scale ranging from *I do not agree at all* (1) to *I completely agree* (7).

#### Intention

Intention was assessed by three items measuring the likelihood of using doping substances in the following 3 months (i.e., “How strong is your intention to use illegal substances to improve your sporting performance or your physical appearance in the next 3 months?”). The responses were recorded on a 5-point Likert scale ranging from *not strong at all* (1) to *very strong* (5).

### Analyses

#### Testing the Measurement Model

**FIGURE 1:**
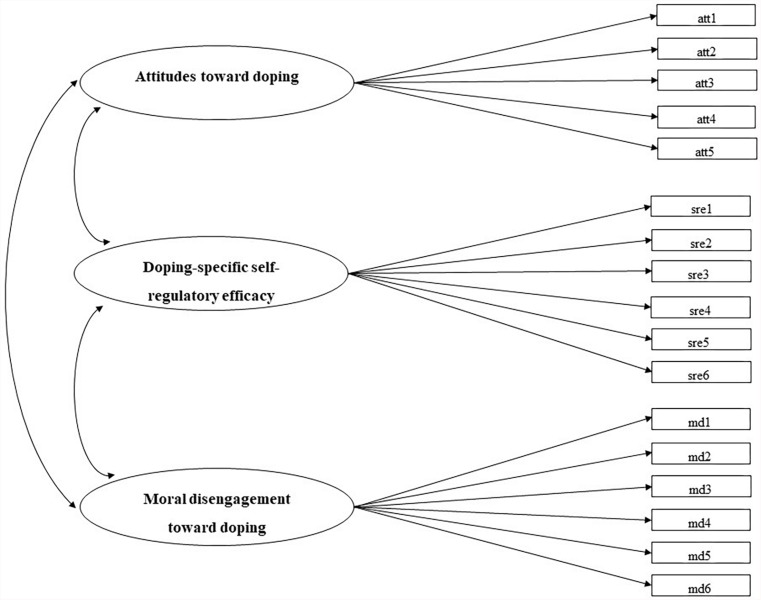
The measurement model for the three instruments used in the study.

Confirmatory factor analyses (CFA) of attitudes, self-regulatory efficacy, and moral disengagement were initially conducted using MPLUS software (Version 8; Muthén and Muthén, 2017). An initial CFA was conducted in order to examine the hypothesis that each set of items measured only one latent factor (i.e., the model implied three factors: attitude, doping-specific self-regulatory efficacy, and moral disengagement) and that the three factors were correlated with each other. The measurement model is displayed in Figure 1. Model parameters were estimated using the maximum likelihood (ML) estimation method, and the quality of the measurement model was examined through multiple fit indices: comparative fit index (CFI), Tucker–Lewis index (TLI), root mean square error of approximation (RMSEA), standardized root mean square residual (SRMR) (Hu and Bentler, 1999), and chi-square (χ^2^)/df ratio (Tabachnick and Fidell, 2006). Cutoff values of 0.90 or above for the CFI were considered indicative of adequate model fit (Marsh et al., 2004). Values of 0.08 or less for the RMSEA and the SRMR were deemed satisfactory for well-fitting models (Marsh et al., 2004). A value of two or less for the χ^2^/df ratio is considered a good indicator of model fit (Tabachnick and Fidell, 2006); however, Kline (1998) suggested that a χ^2^/df ratio of three or less is also a reasonably good indicator of model fit.

#### Evaluating Measurement Invariance Across Genders and Countries and Estimating Latent Mean Differences

Subsequently, in order to test the hypothesis of measurement invariance across genders and countries (i.e., that measurement structure of the instruments applies equally well to males and females, as well as to each country), a second series of CFAs of the model were performed. In line with the literature (Byrne, 2008), these multigroup CFAs tested the configural equivalence (to ascertain that the number of factors and their loading pattern are invariant across groups), the measurement or metric equivalence (to ascertain that all the factor loadings are invariant across groups), and the scalar equivalence (to ascertain that all the item intercepts are invariant across groups). The fit of the models was compared using the change in CFI values (ΔCFI ≤ 0.01) according to Cheung and Rensvold (2002). In addition, in order to test differences with regard to the factors considered in the study, latent mean differences were estimated across genders and countries for each of the three factors—attitudes, self-regulatory efficacy, and moral disengagement—by fixing the latent factor means for one group (i.e., the reference group) to zero and freely estimating the latent factor means for the other groups. Latent mean differences were estimated separately for genders and countries.

#### Testing the Structural Equation Model

In order to evaluate the criterion and predictive validity of the measures, we tested the hypothesis that adolescents’ attitudes, self-regulatory efficacy, and moral disengagement were predictive of doping intention in a Structural Equation Model (SEM). As for the CFA, model parameters were estimated using the ML estimation method using MPLUS software (Version 8; Muthén and Muthén, 2017), and the quality of the structural model was examined through multiple fit indices: CFI, TLI, RMSEA, SRMR (Marsh et al., 2004), and χ^2^/df ratio (Tabachnick and Fidell, 2006). The same cutoff values used for the evaluation of the model fit of the CFA were used for the SEM.

## Results

### Descriptive Statistics, Reliability, and Correlations

Table 1 shows descriptive statistics, reliability, and zero-order correlations of all the key variables of the study for the total sample. Cronbach’s alpha coefficients indicated an acceptable to good reliability for the scores on all of the scales. Zero-order correlations showed that doping intention was positively correlated with attitudes and with moral disengagement toward doping and negatively correlated with doping-specific self-regulatory efficacy.

### The Measurement Invariance of the Scales Across Genders and Countries

Configural, metric, and scalar measurement invariance of the model was tested across genders and countries. Table 2 shows the goodness-of-fit indexes for all the models tested. With respect to measurement invariance across genders, the comparison of the configural invariance model with the metric invariance model showed that the difference in the CFI was smaller than the cutoff criterion (ΔCFI = 0.009), providing support for the metric invariance of the scales across genders. Furthermore, the comparison of the metric invariance model with the scalar invariance model provided support for the full scalar invariance of the scales across genders (ΔCFI = 0.005). With regard to measurement invariance across countries, the comparison of the configural invariance model with the metric invariance model confirmed the metric invariance of the scales across Italy, Romania, and Turkey as the difference in the CFI was smaller than the cutoff criterion (ΔCFI = 0.005). However, the comparison between the scalar invariance model and the metric invariance model indicated that there was not a complete scalar equivalence. In fact, when the model was revised to include the constraints of the item intercepts, the difference in the CFI was bigger than the cutoff criterion. When we examined the modification indices obtained, we found that for six of the items, the intercepts were not statistically equivalent across countries. Accordingly, the equality constraints for these items were released (i.e., the item intercepts were freely estimated) and a second multigroup CFA was then performed. The results of this analysis suggested a partial scalar equivalence across the three national samples, as indicated by an improved ΔCFI (0.008). Table 3 shows the standardized factor loadings and the reliability for each group—for males and females and for Italian, Romanian, and Turkish—as well as the items that were non-invariant across the countries.

**TABLE 2:** Measurement invariance across gender and country.

	**χ^2^**	***df***	**CFI**	**TLI**	**RMSEA**	**SRMR**	**χ^2^/*df***	**Models compared**	**ΔCFI**
**Gender**									
Configural invariance	431.527	232	0.908	0.892	0.066	0.060	1.860		
Metric invariance	466.737	246	0.899	0.888	0.067	0.071	1.897	Metric against configural	0.009
Scalar invariance	490.595	260	0.894	0.889	0.067	0.073	1.886	Scalar against metric	0.005
**Country**									
Configural invariance	524.070	348	0.920	0.906	0.062	0.070	1.505		
Metric invariance	562.137	376	0.915	0.908	0.061	0.077	1.495	Metric against configural	0.005
Scalar invariance	720.767	404	0.856	0.855	0.077	0.093	1.784	Scalar against metric	0.059
Partial scalar invariance	602.178	398	0.907	0.905	0.062	0.079	1.513	Scalar against metric	0.008

*CFI, comparative fit index; TLI, Tucker–Lewis index; RMSEA, root mean square error of approximation; SRMR, standardized root mean square residual; df, degrees of freedom.*

**TABLE 3:** Standardized factor loadings and internal reliability for the three instruments across genders and across the three countries participating in the study (i.e., Italy, Romania, and Turkey).

	**Multigroup-CFA factor loadings**				
	**Gender**		**Countries**		
	**F**	**M**	**IT**	**RO**	**TU**
(1) Attitudes toward doping					
The use of illegal substances to improve sporting performance or physical appearance would be for you:					
1. Useless/useful	0.64	0.57	0.49	0.76	0.74
2. Foolish/wise	0.57	0.53	0.47	0.68	0.73
3. Undesirable/desirable	0.73	0.81	0.75	0.67	0.74
4. Negative/positive	0.77	0.80	0.83	0.67	0.82
5. Harmful/beneficial	0.79	0.82	0.80	0.81	0.82
Cronbach’s alpha	0.82	0.81	0.74	0.85	0.87
(2) Doping-specific self-regulatory efficacy					
You would be able to resist the temptation to use doping substances					
1. …even in the case you have a fall in performance	0.65	0.60	0.61	0.52	0.67
2. …to have a physique more appreciated by others, even if nobody will ever know it	0.60	0.67	0.76	0.61	0.62
3. …to make your body closer to how you would like it	0.65	0.76	0.75	0.64	0.70
4. …to achieve faster results, even if nobody will ever know it	0.76	0.77	0.82	0.65	0.79
5. …despite other people suggest me to do it	0.73	0.70	0.75	0.63	0.70
6. …to improve in the sport you practice, even if you know that wouldn’t have any side effects	0.75	0.76	0.80	0.67	0.74
Cronbach’s alpha	0.84	0.86	0.88	0.80	0.85
(3) Moral disengagement toward doping					
How much do you agree with each of these statements?					
1. Compared to the damaging effects of alcohol and tobacco, the use of illicit substances is not so bad	0.57	0.52	0.44	0.52	0.52
2. It is not right to condemn those who use illicit substances to improve their body, since many people do the same	0.50	0.48	0.51	0.49	0.49
3. Doping use is just another good way to “maximize its potential”	0.55	0.47	0.37	0.48	0.53
4. There is no reason to punish people who use illicit substances to improve their physical appearance, after all, no one gets hurt	0.56	0.51	0.49	0.54	0.55
5. People who use illicit substances in sport are not to blame, to blame are those who expect too much from him	0.54	0.53	0.51	0.50	0.55
6. To overcome their own limitation, it is reasonable to use also illicit substances	0.79	0.75	0.77	0.70	0.78
Cronbach’s alpha	0.76	0.68	0.65	0.72	0.72

*F, female; M, male; IT, Italy; RO, Romania; TU, Turkey; CFA, confirmatory factor analysis. All the factor loadings are statistically significant (*p* < 0.001). The items for which the intercepts were non-invariant between the countries are underlined.*

### Differences in Attitudes, Self-Regulatory Efficacy, and Moral Disengagement Between Genders and Countries

Table 4 shows the results of the analysis of the latent mean differences across genders and countries with regard to the factors considered in the study. Male students showed lower levels of self-regulatory efficacy than females, but there were no statistically significant differences between males and females regarding attitudes and moral disengagement, although males tended to have more positive attitudes toward doping (*p* = 0.08). Both Romanian and Turkish students showed higher levels of moral disengagement and lower levels of self-regulatory efficacy than Italian students. There were no statistically significant differences between the three groups with regard to attitudes, although Romanian students tended to have more positive attitudes toward doping than Italian students, but this finding did not reach a level of statistical significance (*p* = 0.06).

**TABLE 4:** Results of the latent factor mean difference tests.

	**Difference tests**		
**Doping-related constructs**	**Gender**	**Countries**	
	**Females versus Males^a^**	**Italians versus Romanians^b^**	**Italians versus Turkish^b^**
Attitudes	0.158	0.234	–0.110
Self-regulatory efficacy	−0.374*	−0.830***	−0.383*
Moral disengagement	0.041	0.553***	0.412***

^*a*^Females are the reference group for the comparison (the latent mean for this group is fixed to be zero). ^*b*^Italians are the reference group for the comparison (the latent mean for this group is fixed to be zero). *p < 0.05; ***p < 0.001

### The Criterion and Predictive Validity of the Three Measures

**FIGURE 2:**
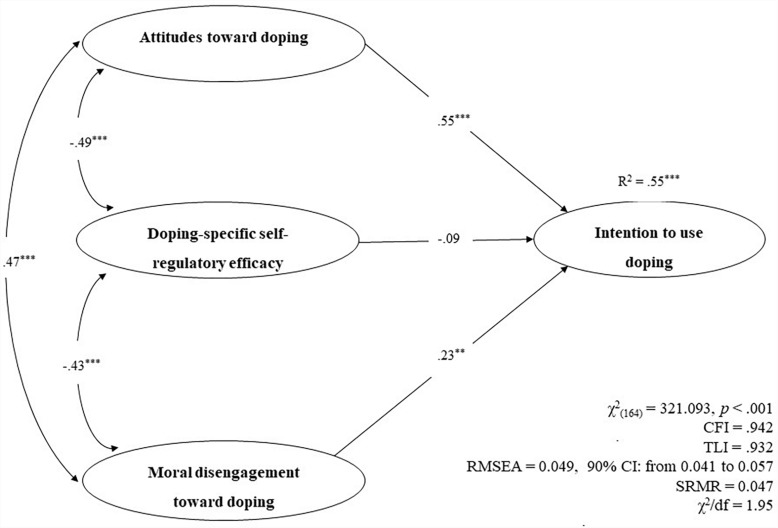
The results of the structural equation model (SEM) in which high-school student’ attitudes, self-regulatory efficacy and moral disengagement predicted doping intention. ***p < 0.001.

As displayed in Figure 2, the results of the SEM analysis met the multiple criteria for adequate model fit, thus supporting the hypothesis that the set of instruments together predicted students’ prospective intentions to use doping. Furthermore, students’ moral disengagement and attitudes uniquely and significantly predicted their intentions to use doping substances. These latter estimates went in the expected directions, suggesting that a greater degree of moral disengagement and more positive attitudes would lead to stronger doping intentions. Although the observed scores of self-regulatory efficacy and intention to use doping are statistically significant and negatively correlated (Table 1), the path between these two variables seemed to be statistically insignificant in the SEM analyses. Therefore, in order to test whether this was a “suppressor effect” due to intercorrelations between the three predictors, a dominance analysis was computed assessing the relative importance of the three regressors in the linear model, using R (Grömping, 2006). The results of this analysis, which was conducted with the R package *relaimpo* using the metric *lmg*, confirmed the contribution of self-regulatory efficacy to the R^2^ increase, therefore confirming the hypothesis of the suppressor effect. Results for the latter analysis are included in the Supplementary Materials.

## Discussion

Pro-doping attitudes, self-regulatory efficacy, and moral disengagement toward doping are self-reported measures that are widely used in the context of doping prevention (Lucidi et al., 2008; Ntoumanis et al., 2014; Kavussanu et al., 2016; Mallia et al., 2016). Those studies conducted hitherto have investigated the psychometric properties of these measures only in the context of sport, and none of them have either analyzed their factorial validity in a sample of non-athletes or tested their measurement invariance across different genders and cultures. The aim of the present study was therefore to test the three-factor structure of the measures in non-athletes, as well as their measurement invariance in males and females and in three different countries: Italy, Romania, and Turkey. Examining whether a set of instruments measuring the determinants of doping is invariant across cultures and genders will allow researchers to properly use and interpret their results. The findings of the CFA provided evidence for the factorial validity of the set of instruments.

Multigroup CFAs conducted on gender and on the three countries supported full configural, metric, and scalar invariance between males and females and full configural, metric, and partial scalar invariance between Italian, Romanian, and Turkish high-school students. Although the full measurement invariance across countries was not achieved, findings may allow appropriate cross-group comparisons (Vandenberg and Lance, 2000). These results suggest that for males and females as well as for Italian, Romanian, and Turkish high-school students, the set of measures has the same structure, with most of the items being equally associated with pro-doping attitudes, self-regulatory efficacy, and moral disengagement toward doping. The scalar invariance across countries and genders allowed us to directly compare the latent means. The findings revealed that girls perceived themselves as significantly more efficacious in resisting social pressure to practice doping than did boys, whereas no significant gender differences were found in moral disengagement and pro-doping attitudes. However, boys expressed more positive attitudes toward doping use than girls did, even though this result did not reach a level of statistical significance. Differences between boys and girls are consistent with previous studies on doping conducted with high-school students (i.e., Lucidi et al., 2008) showing that girls are better able to deal with personal or interpersonal pressure than are boys. Regarding differences between countries, the results show that both Romanian and Turkish students had significantly higher levels of moral disengagement and significantly lower levels of self-regulatory efficacy than Italian students. When asked about their level of self-justification of doping conducts and their capability to resist the social pressure that encourage doping, Romanian and Turkish high-school students reported that they lowered their personal moral standards to a greater extent and that they felt higher levels of self-efficacy, as compared to their Italians peers. Mallia et al. (2016) found similar differences between countries, with German athletes considering themselves more able to resist social pressure with regard to doping use than did Greek and Italian athletes, and with Greek athletes reporting that they suspended their personal moral standards to a greater extent than did Italian and German peers. To our knowledge, no previous studies exist that compare Italian, Romanian, and Turkish high-school students concerning these factors. Differences in perceived self-efficacy and moral disengagement toward doping may simply reflect cultural differences and national customs or traditions. Further studies are needed in order to better explain cross-national differences regarding self-efficacy beliefs and moral disengagement.

Finally, the SEM findings of the present study supported the hypotheses that the measures had criterion and predictive validity. However, it is important to note that, contrary to our hypothesis, students’ perceptions of their self-regulatory efficacy to resist external pressure toward doping did not uniquely predict adolescents’ intentions to practice doping. This finding was probably due to the intercorrelations between self-regulatory efficacy and the other two predictors of intention as the dominance analysis confirmed that self-regulatory efficacy was a significant predictor of intention in a multiple regression model.

It is important to note some limitations of our results. First, the sample size was limited. Second, the study included only participants from Italy, Romania, and Turkey. It would be interesting to replicate this study in a larger sample size and in other countries. Despite these limitations, our results suggest that the instruments examined are reliable and can be used in high-school students to measure their positive attitudes, self-regulatory efficacy, and moral disengagement toward doping.

## Conclusion

In recent years, interest has been growing in doping research. Various studies have shown that the use of illegal PAES is not limited to athletes (Mallia et al., 2013), especially with regard to adolescents (Barkoukis et al., 2016). Most of the research has identified positive attitudes toward doping, morality, and self-efficacy to resist doping as the strongest psychological predictors of doping intentions and behaviors (Ntoumanis et al., 2014). It is therefore fundamental to have a valid instrument for the screening and evaluation of these constructs in order to prevent doping among young people. Furthermore, doping use is influenced by a combination of factors, such as personal characteristics and social contexts (Ntoumanis et al., 2014), that may be associated with different countries or individual experiences. It is therefore important to adopt measures that can be utilized regardless of the national context or characteristics specific to the individual. Our findings suggest that the measures investigated are invariant across genders and partially invariant across countries, so that the reports of males or females, as well as of Italian, Romanian, and Turkish students on these constructs can be meaningfully compared. It seems safe to conclude that the instruments analyzed can be reliably used with high-school students to measure their positive attitudes toward doping, self-regulatory efficacy, and moral disengagement, thereby helping teachers and health practitioners to predict young people’s use of doping or intentions to resort to doping in the future. Finally, the instruments may also be used to measure the effects of specific school-based interventions aimed at preventing the practice of doping (Lucidi et al., 2017).

## Data Availability Statement

The datasets generated for this study are available on request to the corresponding author.

## Ethics Statement

All procedures performed in studies involving human participants were in accordance with the ethical standards of the institutional and/or national research committee and with the 1964 Helsinki declaration and its later amendments or comparable ethical standard. All participants were in no risk of physical or emotional pressure. Written informed consent was obtained from all individual participants included in the study and the results were disseminated only anonymously. None of the participants were patients, or persons with disabilities.

## Author Contributions

LG made the greatest contribution to the manuscript, performing the statistical analyses, drafting the work, and contributing to all the steps of the work. EC revised the statistical analyses. SM, AC, FG, and MC revised the first draft of the manuscript. FL and FA revised the manuscript and monitored all the process providing scientific and theoretical contribution. All authors approved the final version of the manuscript and agreed to be accountable for all aspects of the work in ensuring that questions related to the accuracy or integrity of any part of the work are appropriately investigated and resolved.

## Conflict of Interest

The authors declare that the research was conducted in the absence of any commercial or financial relationships that could be construed as a potential conflict of interest.
