# Supplementary material for: Doping Use in High-School Students: Measuring Attitudes, Self-Efficacy, and Moral Disengagement Across Genders and Countries
Source: Front Psychol. 2020 Apr 28;11:663. doi: 10.3389/fpsyg.2020.00663 (PMC7198734; doi:10.3389/fpsyg.2020.00663)
Supplement: Supplementary file 1 [file Data_Sheet_1.docx]

# Appendix A. The measures used in this study. Italian version.

| **Attitudes toward doping** | |
| --- | --- |
| L'uso di sostanze illegali per migliorare le prestazioni sportive o l'aspetto fisico sarebbe per te: | |
|  | Inutile/utile. |
|  | Incosciente/saggio. |
|  | Indesiderabile/desiderabile. |
|  | Negativo/positivo. |
|  | Dannoso/salutare. |
| **Doping-specific self-regulatory efficacy** | |
| Quanto sei capace di resistere alla tentazione di usare sostanze illegali… | |
| 1. | … anche quando hai un calo di forma. |
| 2. | … per avere un fisico più apprezzato dagli altri, anche se nessuno lo saprà mai. |
| 3. | … per rendere il tuo corpo più vicino a come lo vorresti. |
| 4. | … per raggiungere risultati più velocemente in un’attività sportiva, anche se nessuno lo saprà mai. |
| 5. | … nonostante i suggerimenti di altre persone. |
| 6. | … per migliorare in uno sport che pratichi, anche se sai che non avresti effetti collaterali. |
| **Moral disengagement toward doping** | |
| Quanto sei d’accordo con le seguenti affermazioni? | |
| 1. | Rispetto ai danni causati dall'alcol e dal tabacco, usare sostanze vietate nello sport non è poi così pericoloso. |
| 2. | Non si può condannare chi usa sostanze proibite per migliorare il proprio fisico, visto che molti lo fanno. |
| 3. | Usare sostanze proibite è un modo per “migliorare al massimo le proprie potenzialità”. |
| 4. | Non si ha motivo di punire chi usa sostanze vietate per migliorare il proprio aspetto fisico, in fondo non si fa male a nessuno. |
| 5. | Chi prende sostanze vietate nello sport non ha colpa, la colpa è di chi si aspetta troppo da lui. |
| 6. | Per superare i propri limiti, è lecito anche servirsi di sostanze proibite. |
