# Supplementary material for: Doping Use in High-School Students: Measuring Attitudes, Self-Efficacy, and Moral Disengagement Across Genders and Countries
Source: Front Psychol. 2020 Apr 28;11:663. doi: 10.3389/fpsyg.2020.00663 (PMC7198734; doi:10.3389/fpsyg.2020.00663)
Supplement: Supplementary file 2 [file Data_Sheet_2.docx]

# Appendix B. The measures used in this study. Romanian version.

| **Attitudes toward doping** | |
| --- | --- |
| Utilizarea substanţelor în scopul îmbunătăţirii performanţei sportive sau a aspectului fizic în  următoarele trei luni ar fi: | |
|  | Inutilă/ folositoare. |
|  | Nesăbuită /înţeleaptă. |
|  | De nedorit/ de dorit. |
|  | Negativă/pozitivă. |
|  | Nocivă /benefică. |
| **Doping-specific self-regulatory efficacy** | |
| În ce măsură te simţi încrezător pentru a evita utilizarea substanţelor ilicite… | |
| 1. | … chiar și atunci când vă simţiţi extenuate fizic. |
| 2. | … pentru a avea un corp pe care alţii îl vor admira, chiar și atunci când nimeni nu ar ști. |
| 3. | … pentru a avea corpul așa cum doriţi. |
| 4. | … pentru a ajunge la rezultate sportive mai repede, chiar și atunci când nimeni nu va ști vreodată. |
| 5. | … în ciuda presiunii exercitate de alţii. |
| 6. | … pentru a vă îmbunătăţi performanţa în sportul pe care îl practicaţi, chiar și atunci când știţi că nu va avea efecte secundare. |
| **Moral disengagement toward doping** | |
| Cât de mult sunteți de acord cu următoarele afirmații? | |
| 1. | În comparaţie cu efectele nocive ale alcoolului și tutunului, utilizarea de substanţe ilicite nu este atât de rea. |
| 2. | Nu este corect să condamnăm pe cei care folosesc substanţe ilicite, pentru a-și îmbunătăţi corpul, deoarece mulţi fac același lucru. |
| 3. | Utilizarea substanţelor ilicite este o modalitate de a "îmbunătăţi potenţialul propriu în cel mai bun mod". |
| 4. | Nu există niciun motiv pentru a pedepsi pe cei care folosesc substanţe ilicite pentru a-și îmbunătăţi aspectul fizic; in fond, nu fac rău nimănui. |
| 5. | Cine folosește substanţe ilicite în sport nu trebuie să fie acuzat; Vina este a celor care așteaptă prea mult de la el sau ea. |
| 6. | Este bine să utilizaţi substanţe ilicite dacă acest lucru vă poate ajuta să depășiţi limitele proprii. |
