# Supplementary material for: Doping Use in High-School Students: Measuring Attitudes, Self-Efficacy, and Moral Disengagement Across Genders and Countries
Source: Front Psychol. 2020 Apr 28;11:663. doi: 10.3389/fpsyg.2020.00663 (PMC7198734; doi:10.3389/fpsyg.2020.00663)
Supplement: Supplementary file 3 [file Data_Sheet_3.docx]

# Appendix C. The measures used in this study. Turkish version.

| **Attitudes toward doping** | |
| --- | --- |
| Spor performansınızı yada fiziksel görünüşünüzü geliştirme amacıyla önümüzdeki üç ayda madde kullanımı: | |
|  | Gereksiz/gerekli. |
|  | Aptalca/akıllıca. |
|  | Sakıncalı/cazip. |
|  | Olumsuz/olumlu. |
|  | Zararlı/faydalı. |
| **Doping-specific self-regulatory efficacy** | |
| Aşağıdaki konularda ne derecede kendinizden emin olabilirsiniz? | |
| 1. | Fiziksel olarak bilmek yeterli hissettiğinizde safra yasadışı maddeler kullanmaktan sakınmak. |
| 2. | Hiç kimsenin asla bilemeyeceği durumlarda bile, başkalarının beğendiği bir vücuda sahip olmak için yasadışı. |
| 3. | Vücudunuzun istediğiniz gibi görünmesi için yasadışı maddeler kullanmaktan sakınmak. |
| 4. | Hiç kimsenin asla bilemeyeceği durumlarda bile, sporda sonuca daha çabuk ulaşmak için yasadışı maddeler kullanmaktan sakınmak. |
| 5. | Başkalarından gelen baskıya rağmen yasadışı maddeler kullanmamak. |
| 6. | Yan etkileri olmayacağını bildiğinizde bile, yaptığınız spordaki performansınızı arttırmak için yasadışı maddeler kullanmaktan kaçınmak. |
| **Moral disengagement toward doping** | |
| Aşağıdaki ifadelere ne kadar katılıyorsunuz? | |
| 1. | Alkol ve tütünün zararlı etkileriyle karşılaştırıldığında, yasadışı maddelerin kullanımı çok da kötü değil. |
| 2. | Bir çok insan aynısını yaptığı için, vücutlarını geliştirmek için yasadışı madde kullananları kınamak doğru değil. |
| 3. | Yasadışı maddelerin kullanımı, kişinin kendi potansiyelini en iyi şekilde geliştirebilmesinin bir yoludur. |
| 4. | Fiziksel görüntülerini geliştirmek için yasadışı madde kullananları cezalandırmak için herhangi bir neden yok; sonuçta kimseye zarar vermiyorlar. |
| 5. | Sporda yasadışı maddeler kullananlar suçlanmamalıdır; esas suç onlardan çok fazla beklentisi olanlarda. |
| 6. | Eğer kişinin limitlerini aşmasına yardımcı olabiliyorsa, yasadışı maddeler kullanmak kabul edilebilir. |
