# Supplementary material for: Doping Use in High-School Students: Measuring Attitudes, Self-Efficacy, and Moral Disengagement Across Genders and Countries
Source: Front Psychol. 2020 Apr 28;11:663. doi: 10.3389/fpsyg.2020.00663 (PMC7198734; doi:10.3389/fpsyg.2020.00663)
Supplement: Supplementary file 4 [file Data_Sheet_4.docx]

# Appendix D. The measures used in this study. English version.

| **Attitudes toward doping** | |
| --- | --- |
| The use of illegal substances to improve sporting performance or physical appearance would be for you: | |
|  | Useless/useful. |
|  | Foolish/wise. |
|  | Undesirable/desirable. |
|  | Negative/positive. |
|  | Harmful/beneficial. |
| **Doping-specific self-regulatory efficacy** | |
| You would be able to resist the temptation to use doping substances… | |
| 1. | … even in the case you have a fall in performance. |
| 2. | … to have a physique more appreciated by others, even if nobody will ever know it. |
| 3. | … to make your body closer to how you would like it. |
| 4. | … to achieve faster results, even if nobody will ever know it. |
| 5. | … despite other people suggest me to do it. |
| 6. | … to improve in the sport you practice, even if you know that wouldn't have any side effects. |
| **Moral disengagement toward doping** | |
| How much do you agree with each of these statements? | |
| 1. | Compared to the damaging effects of alcohol and tobacco, the use of illicit substances is not so bad. |
| 2. | It is not right to condemn those who use illicit substances to improve their body, since many people do the same. |
| 3. | Doping use is just another good way to "maximize its potential". |
| 4. | There is no reason to punish people who use illicit substances to improve their physical appearance, after all, no one gets hurt. |
| 5. | People who use illicit substances in sport are not to blame, to blame are those who expect too much from him. |
| 6. | To overcome their own limitation, it is reasonable to use also illicit substances. |
