# Supplementary material for: Doping Use in High-School Students: Measuring Attitudes, Self-Efficacy, and Moral Disengagement Across Genders and Countries
Source: Front Psychol. 2020 Apr 28;11:663. doi: 10.3389/fpsyg.2020.00663 (PMC7198734; doi:10.3389/fpsyg.2020.00663)
Supplement: Supplementary file 5 [file Data_Sheet_5.docx]

# Results of the dominance analysis

Call:

lm (formula = Intention ~ Attitudes + Selfeffic + Moraldis)

Residuals:

Min 1Q Median 3Q Max

-1.80669 -0.35397 -0.05292 0.18244 2.80623

Coefficients:

Estimate Std. Error t value Pr(>|t|)

(Intercept) 0.69610 0.18822 3.698 0.000249 ***

Attitudes 0.39115 0.03728 10.492 < 2e-16 ***

Selfeffic -0.06735 0.02269 -2.968 0.003188 **

Moraldis 0.20127 0.04139 4.862 1.7e-06 ***

---

Signif. codes: 0 ‘***’ 0.001 ‘**’ 0.01 ‘*’ 0.05 ‘.’ 0.1 ‘ ’ 1

Residual standard error: 0.6361 on 379 degrees of freedom

(19 observations deleted due to missingness)

Multiple R-squared: 0.4103, Adjusted R-squared: 0.4057

F-statistic: 87.91 on 3 and 379 DF, p-value: < 2.2e-16

Response variable: Intention

Total response variance: 0.6809073

Analysis based on 383 observations

3 Regressors:

Attitudes Selfeffic Moraldis

Proportion of variance explained by model: 41.03%

Metrics are normalized to sum to 100% (rela=TRUE).

Relative importance metrics:

lmg

Attitudes 0.6044004

Selfeffic 0.1725051

Moraldis 0.2230945
